# Role of LMCD1 in the Long-Term Effects of Angiotensin II in Vascular Smooth Muscle Cells

**DOI:** 10.3390/ijms26094053

**Published:** 2025-04-25

**Authors:** Janka Borbála Gém, Kinga Bernadett Kovács, Szilvia Barsi, Saba Hadadnejadtehrani, Amir Damouni, Gábor Turu, András Dávid Tóth, Péter Várnai, László Hunyady, András Balla

**Affiliations:** 1 Department of Physiology, Faculty of Medicine, Semmelweis University, Tűzoltó utca 37-47, 1094 Budapest, Hungary; gem.janka.borbala@semmelweis.hu (J.B.G.); kovacs.kinga2@semmelweis.hu (K.B.K.); saba.hadadnejadtehrani@stud.semmelweis.hu (S.H.); amir.damouni@stud.semmelweis.hu (A.D.); turu.gabor@semmelweis.hu (G.T.); varnai.peter@semmelweis.hu (P.V.); hunyady.laszlo@semmelweis.hu (L.H.); 2 Institute of Molecular Life Sciences, Centre of Excellence of the Hungarian Academy of Sciences, HUN-REN-SU Research Centre of Natural Sciences, Magyar tudósok körútja 2, 1117 Budapest, Hungary; toth.andras1@semmelweis.hu; 3 Department of Internal Medicine and Haematology, Semmelweis University, 1088 Budapest, Hungary; 4 HUN-REN-SU Molecular Physiology Research Group, Hungarian Research Network and Semmelweis University, 1094 Budapest, Hungary

**Keywords:** Angiotensin II (AngII), LIM and cysteine-rich domains 1 (LMCD1), mitogen-activated protein kinase (MAPK), type 1 angiotensin receptor (AT1-R), vascular smooth muscle cell (VSMC)

## Abstract

Excessive activity of the hormone angiotensin II (AngII) is known to contribute to the pathogenesis of multiple cardiovascular diseases, including atherosclerosis, vascular remodeling, and hypertension, primarily through inducing gene expression changes. In this study, we identified LMCD1 (LIM and cysteine-rich domains 1, also known as Dyxin), primarily recognized as a transcription co-factor involved in various oncogenic processes, cardiac hypertrophy, and vascular remodeling, as a potential key factor in AngII-mediated effects in vascular smooth muscle cells (VSMCs). We demonstrated that AngII upregulates *LMCD1* expression in primary rat VSMCs through type 1 angiotensin receptor (AT1-R) activation, leading to calcium signaling and p38 MAPK pathway activation. Additionally, we also demonstrated in A7r5 vascular smooth muscle cells that LMCD1 protein overexpression results in enhanced cell proliferation and cell migration. Our findings provide insights into the mechanisms by which AngII mediates changes in *LMCD1* expression. The elevated expression of LMCD1 enhanced the cell proliferation and migration in VSMCs in vitro experiments, suggesting that LMCD1 may be an important factor in vascular remodeling and the pathogenesis of severe cardiovascular diseases. These results raise the possibility that LMCD1 could be a promising pharmacological target in the cardiovascular dysfunctions associated with AT1-R overactivation.

## 1. Introduction

Octapeptide hormone angiotensin II (AngII) plays a pivotal role as the primary effector molecule of renin–angiotensin–aldosterone system (RAAS). Its effects, both short and long-term, are primarily mediated through the binding to type 1 angiotensin receptor (AT1-R), a member of the G-protein-coupled receptor (GPCR) superfamily. AT1-R mainly acts via G_q/11_-coupled signal transduction pathway, leading to the activation of phospholipase Cβ, which cleaves phosphatidylinositol-4,5-bisphosphate (PtdIns(4,5)*P*_2_) into diacylglycerol (DAG) and inositol-1,4,5-trisphosphate (Ins(1,4,5)*P*_3_), which serve as second messenger molecules. DAG activates protein kinase C (PKC), initiating numerous intracellular pathways, while Ins(1,4,5)*P*_3_ triggers the release of Ca^2+^ from the endoplasmic reticulum (ER), thereby inducing calcium signaling pathways [1]. In addition to these mechanisms, AngII-stimulated AT1-R can also activate other G proteins, such as G_12/13_ or G_i/o_ [2,3]. Furthermore, AT1-R also can initiate G-protein-independent signaling, primarily through β-arrestin activation [4]. 

It is well established that the primary short-term effect of AngII in vascular smooth muscle cells (VSMC) is the induction of vasoconstriction through G_q/11_ mediated Ca^2+^ release. This rapid regulation of arterial blood pressure represents a significant mechanism in rapid cardiovascular adaptations [1]. Additionally, AngII stimulates aldosterone release from the adrenal gland granulosa cells and induces a sense of thirst in the central nervous system. As such, AngII emerges as a crucial regulator of the human body’s electrolyte and fluid homeostasis [5]. Moreover, the AngII also elicits long-lasting effects on the gene expression levels of numerous proteins, leading to the proliferation and migration of VSMCs, as well as the remodeling of blood vessels [1]. Prolonged and exaggerated AT1-R activation is associated with various pathological outcomes, including vascular remodeling, enhancing atherosclerosis formation, and the development of hypertension. Consequently, these long-term effects of AngII action significantly contribute to the progression of serious cardiovascular diseases, such as heart failure or acute coronary syndrome [6]. The activation of the mitogen-activated protein kinase (MAPK) pathways plays a significant role in the development of long-lasting and often pathological effects of AngII [7]. AT1-R-mediated activation of various MAPKs in VSMCs is predominantly influenced by the transactivation of receptor tyrosine kinases, particularly the epidermal growth factor receptor (EGFR) [1,8,9]. Additionally, AT1-R-mediated transactivation of other receptor tyrosine kinases, such as the platelet-derived growth factor receptor (PDGFR) and insulin-like growth factor 1 receptor (IGF1R), has also been documented [10,11]. This transactivation is promoted by matrix-metalloprotease-mediated shedding of heparin-binding epidermal growth factor-like growth factor (HB-EGF) [12]. 

LIM and cysteine-rich domains 1 (LMCD1; also known as Dyxin) is a member of the LIM protein family [13]. LIM proteins have diverse roles in cellular functions, primarily regulating gene expression, cytoskeletal remodeling, cell adhesion, cell motility, and signal transduction [14]. They are recognized as modulators of GATA function, especially in cardiac, pulmonary, and hematopoietic tissues [15,16]. Structurally, LMCD1 contains two LIM domains at the C-terminus, a cysteine-rich domain at the N-terminus, and a PET (Prickle, Espinas, and Testin) domain in the central region [13]. LIM domains contain conserved zinc-binding residues that can establish zinc-finger structures. Research on LMCD1 functions is still limited compared to other LIM proteins, but it has been identified as a repressor for GATA6, inhibiting its DNA binding in lung epithelium, cardiac myocytes, and vascular smooth muscle cells [17,18]. In the heart, LMCD1 has been linked to provoke cardiac hypertrophy via activation of calcineurin in mice, with increased cell growth and fibrosis observed in cardiac myocytes [19,20]. It has been recently reported that LMCD1 is involved in renal fibrosis [21] and in the pathogenesis of lung diseases in systemic sclerosis [17]. In the human aortic smooth muscle cell line, it was observed that thrombin significantly increased *LMCD1* expression through G_q/11_ activation, promoting vascular smooth muscle proliferation and atherogenesis [18]. Given the limited information about LMCD1 in both the physiological and pathophysiological actions of AngII —particularly in its primary target cells, vascular smooth muscle cells—we aimed to characterize the changes in LMCD1 expression in response to AngII stimulation, as well as its localization and functional effects in rat VSMCs.

As previously mentioned, AngII stimulation not only causes contraction in VSMCs but also leads to long-term changes in gene expression. While we have a comprehensive understanding of these mechanisms, the results were primarily derived from studies using immortalized cell cultures or VSMCs maintained for extended periods. It is well-known that primary VSMCs undergo increasing phenotypic changes with each passage. These changes typically start to manifest after 7–9 days in culture. To provide conditions that closely mirror in vivo circumstances, we used primary rat aortic VSMCs, limiting our experiments using cells in their third passage at the latest, thus avoiding aleatory phenotypic changes. These cell cultures are expected to report results that more sensibly reflect in vivo conditions. 

The current study was undertaken to investigate the potential role of LMCD1 in response to AngII stimulation. Firstly, we investigated the expression and effects of LMCD1 in this study in rat VSMCs. We found that both gene and protein expressions of LMCD1 were elevated consistently in response to AngII stimulation, which indicates that LMCD1 can be an important effector in the long-term action of AngII on VSMCs. Secondly, we demonstrated that overexpression of LMCD1 protein in the A7r5 vascular smooth muscle cell line leads to increased cell proliferation and cell migration. Taken together, our data suggest that AngII-induced LMCD1 protein overexpression is an important factor mediating long-term effects, not only in the heart [19] but also within the vasculature, contributing to the broader impact on the cardiovascular system.

## 2. Results

### 2.1. Differential Expression Analysis Reveals AngII-Induced Upregulation of LMCD1

**Figure 1:**
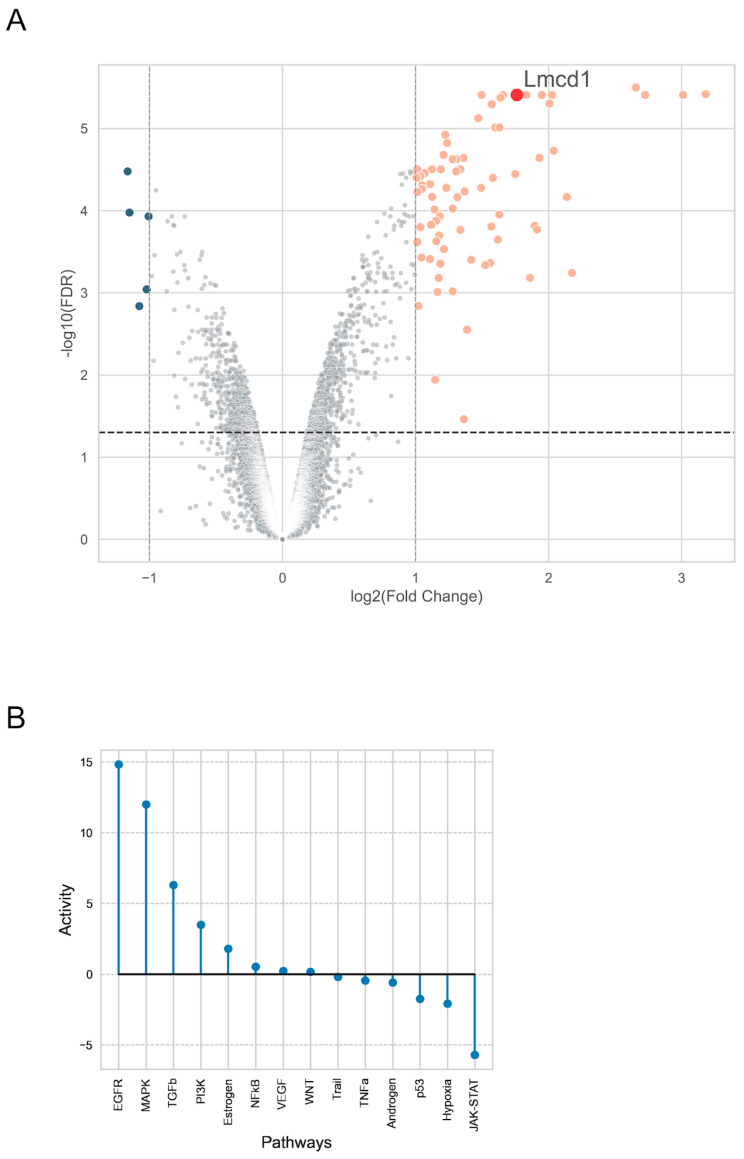
AngII-induced gene expression changes in VSMCs. (**A**) The volcano plot illustrates differential gene expression in VSMCs after 2 h of AngII stimulation compared to the vehicle-treated control group. The *x*-axis represents the log2 fold change in gene expression, while the *y*-axis shows the −log10 of the false discovery rate (FDR). Genes highlighted in color exhibit a log2 fold change greater than 1, either upregulated or downregulated. *LMCD1* is specifically labeled. The horizontal dotted line marks the significance threshold, where FDR < 0.05. (**B**) PROGENy pathway analysis of the AngII-induced gene expression signature. For the PROGENy pathways (*x*-axis), activity scores were calculated (*y*-axis) using a multivariate linear model.

We investigated the genes upregulated in VSMCs in response to AngII stimulation, aiming to better understand the cell responses induced by AngII. To evaluate changes in gene expression, we performed an Affymetrix microarray analysis on primary rat aortic vascular smooth muscle cells after a 2-h stimulation with 100 nM AngII. Interestingly, *LMCD1* was among the most upregulated genes in the AngII-treated samples, with a log2-fold change in 1.76 and a highly significant differential expression with an adjusted *p*-value of 3.9 × 10^−6^, as shown in Figure 1A. We calculated pathway activities from the differential expression signature using the PROGENy (Pathway RespOnsive GENes), which contains a curated collection of pathways and their responsive genes. The analysis revealed that AngII treatment significantly increased the activity of the EGFR, MAPK, and TGFβ pathways (Figure 1B). Notably, *LMCD1* is a responsive gene of the TGFβ pathway.

### 2.2. Validation of Affymetrix Array Results and Evaluation of Time Dependence of LMCD1 Expression in Response to AngII Stimulation in VSMCs

**Figure 2:**
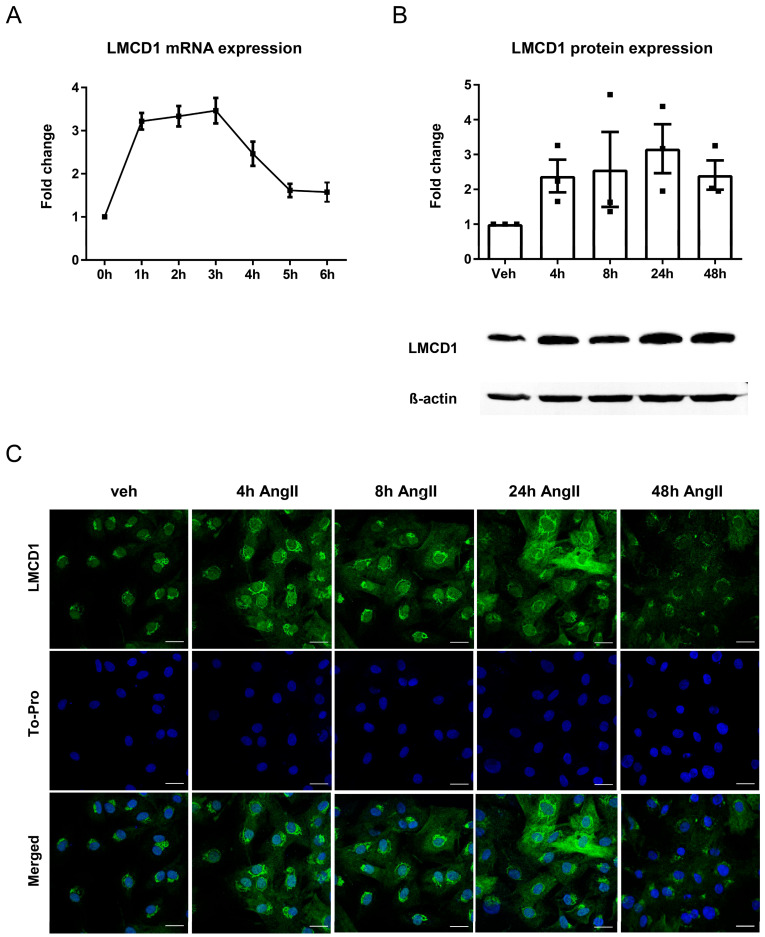
Time kinetics of LMCD1 gene and protein expression. VSMCs were serum-starved overnight and then treated with 100 nM AngII. (**A**) VSMCs were stimulated with AngII every hour for 6 h, and then mRNA was isolated from samples. The expression of *LMCD1* was measured using quantitative real-time PCR, with standardization against the *GAPDH* housekeeping gene. Mean values ± SE are presented *(n* = 5). (**B**) LMCD1 protein expression was measured using Western blot assay. Cells were treated with AngII for various time periods between 0 and 48 h. The membranes were labeled with either anti-LMCD1 primary antibody or anti-beta actin (loading control). HRP-linked or fluorescent secondary antibodies were used for detection (a representative immunoblot record is shown in the upper part of the graph). Protein levels were normalized to unstimulated (0 h) samples. Mean values ± SE are shown *(n* = 3). (**C**) Cells were treated with AngII for various time periods between 0 and 48 h. Samples were fixed with paraformaldehyde and stained with an anti-LMCD primary antibody and an AlexaFluor488-labeled secondary antibody (green). Nuclei of cells were stained with To-Pro reagent (blue). Immunofluorescent signals were examined using a Zeiss LSM710 confocal laser-scanning microscope. Scale bars represent 25 µm.

We confirmed the results of the transcriptome analysis regarding *LMCD1* gene expression changes in response to AngII stimulation using quantitative real-time PCR measurements. This method not only validated the gene chip results but also allowed us to determine the time kinetics of *LMCD1* gene expression in response to AngII. As shown in Figure 2A, the *LMCD1* level increased approximately three-fold compared to the control level one hour after the AngII stimulation and significantly decreased 4 h after the hormonal stimulation. Based on this observation, subsequent experiments investigating AngII-induced *LMCD1* gene expression were performed with 2-h incubation periods with AngII. Time kinetics of AngII-mediated LMCD1 protein expression were quantified with Western blot determinations (Figure 2B, upper and lower panels). The results indicate that LMCD1 protein levels peak around 24 h after AngII stimulation and significantly decrease after 48 h. Similar results were obtained using immunofluorescence microscopy, showing the most intense presence of LMCD1 protein in VSMCs stimulated with AngII for 24 h (Figure 2C).

### 2.3. LMCD1/Dyxin Is Primarily Localized in the Nucleus and the Golgi Apparatus

**Figure 3:**
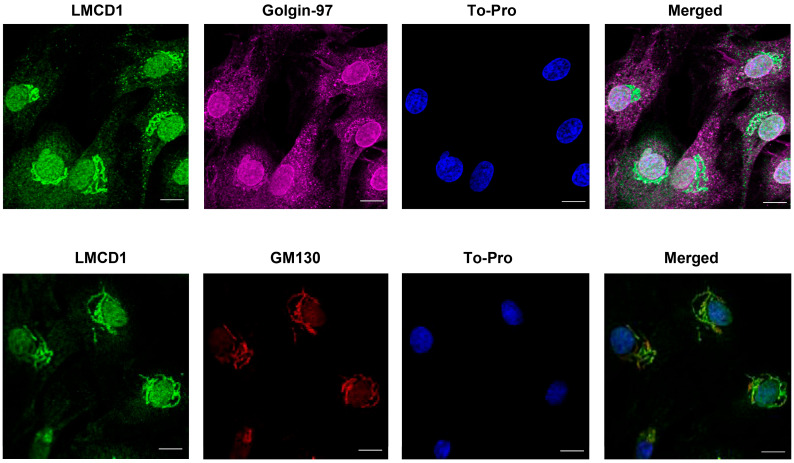
Intracellular localization of LMCD1. Serum-deprived VSMCs were treated with 100 nM AngII for 24 h. LMCD1 was detected using anti-LMCD1 primary antibody and AlexaFluor488 secondary antibody (green). Cell nuclei were labeled with To-Pro reagent (blue). The trans-Golgi apparatus was labeled with an anti-Golgin-97 antibody (magenta); an anti-GM130 antibody was used to label cis-Golgi (red). Immunofluorescent signals were examined using a Zeiss LSM710 confocal laser-scanning microscope. Scale bars represent 10 µm.

Based on the literature data, LIM proteins can be found in both the cytoplasm and the nucleus, allowing these proteins to interact with numerous target proteins, including kinases, cytoskeletal proteins, and transcription factors [14,16]. To determine the exact intracellular localization of LMCD1 protein in VSMCs, immunofluorescent staining was performed on VSMCs stimulated with AngII for 24 h, the time-point at which the AngII-induced expression was the most intense. Immunostaining of LMCD1 revealed nuclear staining and a pattern resembling typical Golgi apparatus protein localization, though its mitochondrial labeling was uncertain. By labeling various subcellular organelles with specific markers, LMCD1 was found to localize predominantly to the nucleus as well as both the trans-Golgi (upper row) and cis-Golgi (lower row) (Figure 3).

### 2.4. Investigation of Signaling Pathways Leading to LMCD1 Expression Increase Initiated by AngII Stimulation

**Figure 4:**
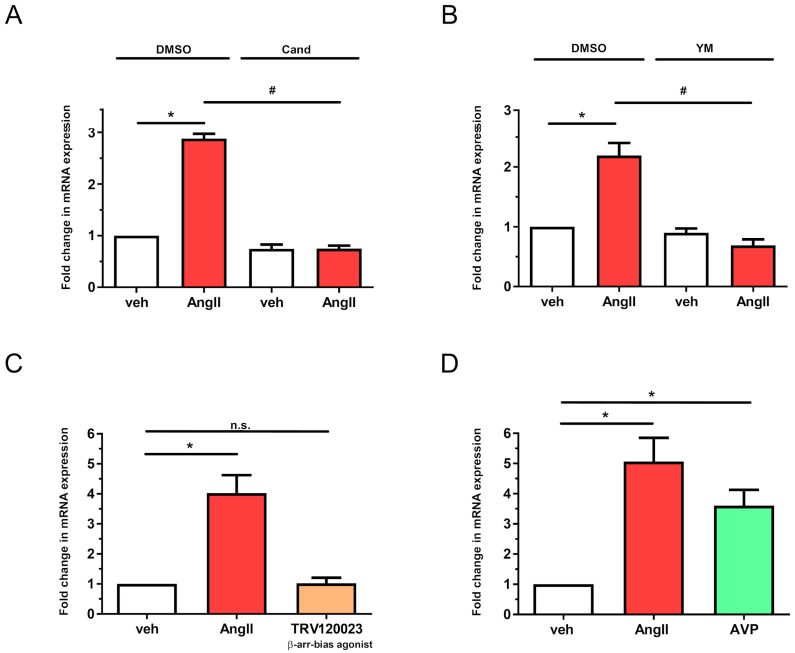
Role of AT1-R and Gq/11 signaling in AngII-mediated *LMCD1* expression. VSMCs were subjected to overnight serum withdrawal prior to pharmacological inhibitor pretreatments and agonist treatments. mRNA levels were measured using quantitative real-time PCR, with *GAPDH* as the housekeeping gene for normalization. (**A**) VSMCs were pretreated with 10 µM Candesartan or DMSO (control) for 30 min, followed by stimulation with vehicle (white columns) or 100nM AngII (red columns) for 2 h (*n* = 4). (**B**) VSMCs were pretreated with 1 µM YM-254890 or DMSO (control) for 30 min, then stimulated with vehicle (white columns) or 100 nM AngII (red columns) for 2 h (*n* = 6). (**C**) VSMCs were incubated with vehicle (white column), 100 nM AngII (red column), or 3 µM TRV120023 (β-arrestin-bias agonist, beige column) for 2 h (*n* = 3). (**D**) VSMCs were stimulated with vehicle (white column), 100 nM AngII (red column), or 1 µM AVP (green column) for 2 h (*n* = 5). Mean values ± SE are shown. Statistical significance was determined using multiple linear regression (**A**,**B**) or 1-way ANOVA (**C**,**D**), with *p* < 0.05 was considered to be statistically significant. Significance compared to vehicle stimulation is indicated by *; significance compared to DMSO-pretreated agonist-induced response is indicated by #; while n.s. indicates not statistically significant (*p* > 0.05).

To characterize the processes leading to *LMCD1* upregulation after AngII treatments, we used different pharmacological inhibitors on primary VSMCs. First, to determine whether AngII upregulates *LMCD1* through AT1-R, we pretreated serum-starved VSMCs with candesartan, a selective AT1-R blocker widely used in antihypertensive therapies [22]. Our results revealed that in 10 µM candesartan-pretreated VSMCs, AngII stimulation had no effect on *LMCD1* gene expression; mRNA levels remained near the baseline (Figure 4A). This finding suggests that AngII regulates *LMCD1* expression predominantly through AT1-R; however, the potential role of the type 2 angiotensin receptor (AT2-R) was not excluded, as a selective AT2-R antagonist was not used.

In VSMCs, AT1-R exerts its most well-known effects through Gq/11 protein, but Gq/11 is not the only G-protein involved in AT1-R signal transduction. YM-254890 (YM) is a selective Gq/11 inhibitor, which we used to determine the importance of the G_q/11_ protein in AngII-mediated *LMCD1* upregulation. In YM-pretreated VSMC, AngII failed to produce its upregulatory effect (Figure 4B), leading to the conclusion that G_q/11_ activation of agonist-bound AT1-R is essential for the *LMCD1* upregulation. 

Since AT1-R primarily functions as a G-protein-coupled receptor, it is expected that AT1-R activation leads to *LMCD1* upregulation via a G-protein coupled manner. However, we must also consider the G-protein-independent signaling capability of AT1-R, notably the β-arrestin-mediated signaling pathway. To assess the possibility that AT1-R activation increases LMCD1 mRNA level through β-arrestin, we used TRV120023, a widely used biased agonist of AT1-R that activates the β-arrestin pathway without significantly activating G-protein-mediated pathways. As shown in Figure 4C, the TRV120023-induced β-arrestin activation did not significantly augment LMCD1 mRNA levels compared to the basal level. This leads us to conclude that the effect of AngII on the *LMCD1* gene expression change is driven by G-protein activation and not β-arrestin-mediated manner. Furthermore, treatment with 100 nM of arginine-vasopressin (AVP), whose receptor (V1 vasopressin 1 receptor; V1R) on VSMCs is also G_q/11_ coupled [23], also enhanced *LMCD1* expression, though less intensely than AngII (Figure 4D). 

**Figure 5:**
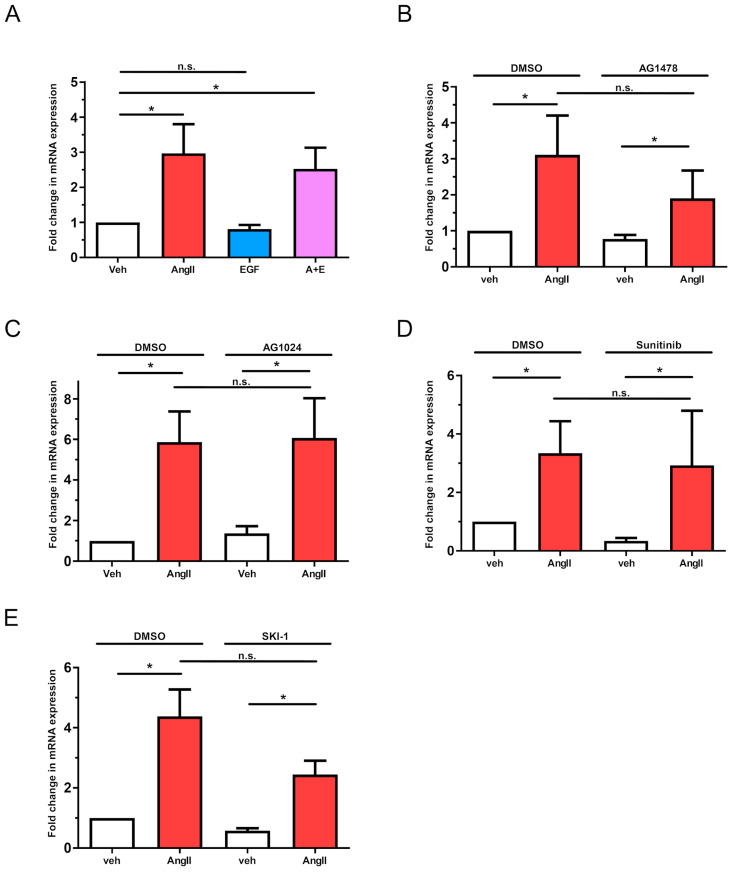
Effects of EGF and tyrosine kinase inhibitors on *LMCD1* expression in vascular smooth muscle cells. (**A**) Serum-deprived VSMCs were treated with either vehicle (white columns), 100 nM AngII (red columns), 50 ng/mL EGF (blue columns), or a combination of 100 nM AngII and 50 ng/mL EGF (purple columns) for 2 h. (**B**–**E**) Serum-starved VSMCs were incubated with either DMSO or (**B**) 1 µM EGFR inhibitor AG1478, (**C**) 1 µM IGF-1R inhibitor AG1024, (**D**) 5 µM of PDGFR and VEGFR inhibitor Sunitinib or (**E**) 10 µM of Src family tyrosine kinase inhibitor SKI-1. *LMCD1* mRNA levels were measured by qRT-PCR and normalized to *GAPDH* as the housekeeping gene, with vehicle-treated samples serving as the baseline. Data are expressed as fold change (mean ± S.E.). Significance was assessed using either one-way ANOVA (**A**) or multiple linear regression (**B**–**E**), with *p* < 0.05 considered to be statistically significant. *: Statistically significant from vehicle stimulation; while n.s. indicates not statistically significant (*p* > 0.05). The values are from three to seven independent experiments (*n =* 3–7).

We also examined the possibility that the transactivation of EGFR or other growth factor receptors might be involved in the AngII-mediated upregulation of *LMCD1*. However, direct stimulation with 50 ng/mL EGF, either alone or in combination with 100 nM AngII, did not result in an enhanced *LMCD1* expression level compared to AngII stimulation alone (Figure 5A). Additionally, various growth factor receptor inhibitors—including the EFGR kinase inhibitor AG1478 (1 µM), the insulin-like growth factor I receptor (IGF-IR) inhibitor AG1024 (1 µM), and the VEGFR and PDGFR inhibitor Sunitinib (5 µM)—did not significantly reduce the AngII-mediated *LMCD1* upregulation (Figure 5B–D). Notably, pretreatment of VSMCs with the Src family tyrosine kinase inhibitor SKI-1 (10 µM) also failed to significantly diminish the AngII-induced increase in *LMCD1* expression (Figure 5E). Together, these data suggest that these receptor- and non-receptor tyrosine kinases do not play key roles in regulating *LMCD1* expression in the long-term effects of AngII.

### 2.5. Investigation of the Role of Calcium Signal in LMCD1 Upregulation

**Figure 6:**
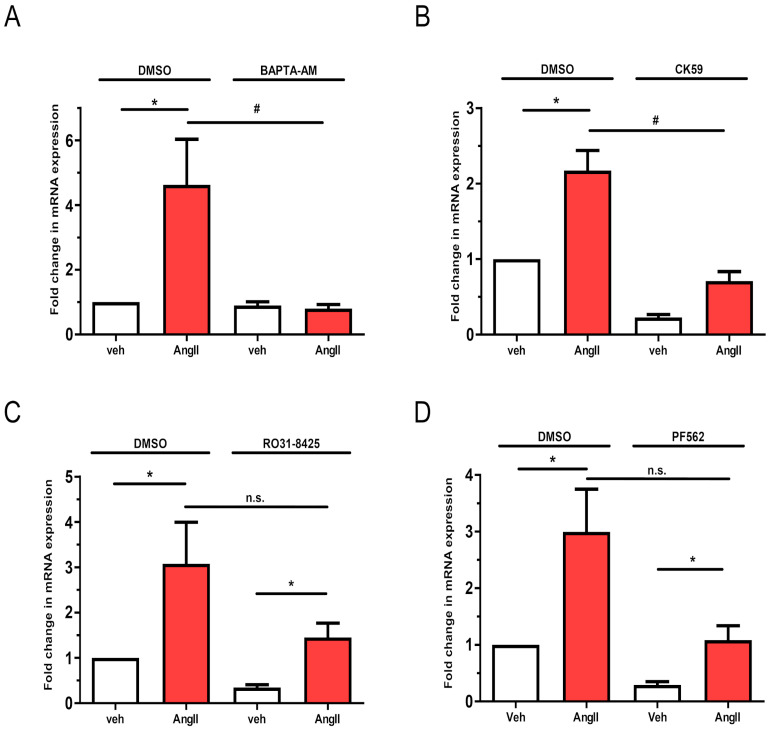
Investigation of calcium signaling and calcium-dependent kinases in AngII-mediated upregulation of *LMCD1* in vascular smooth muscle cells. Seum-deprived VSMCs were pretreated with either DMSO as a control or with one of the following inhibitors: (**A**) 50 μM BAPTA-AM for 10 min, (**B**) 50 μM CamKII inhibitor CK59 for 30 min, (**C**) 1 μM PKC inhibitor RO-31-8425 for 10 min, or (**D**) 1 μM PF-562271 Pyk2 inhibitor. After inhibitor pretreatment, cells were stimulated with either vehicle (white columns) or 100 nM AngII (red columns) for 2 h. mRNA was then isolated and converted to cDNA, and *LMCD1* expression levels were measured by qRT-PCR and normalized to the *GAPDH* housekeeping gene. Data are shown as values normalized to DMSO vehicle samples (mean ± S.E.). Statistical significance was determined using multiple linear regression, with *p* < 0.05 considered to be statistically significant. Significance compared to vehicle stimulation is indicated by *; significance compared to DMSO-pretreated agonist-induced response is indicated by #; while n.s. indicates not statistically significant (*p* > 0.05). Values represent three to five independent experiments (*n =* 3–5).

Our data revealed that *LMCD1* upregulation is mainly associated with G_q/11_ protein activation of AT1-R (Figure 4B). The α subunit of G_q/11_ primarily exerts its effects through Ins(1,4,5)*P*_3_-mediated Ca^2+^ release and/or protein kinase C (PKC), activated by DAG [1]. To assess the Ca^2+^ dependence of *LMCD1* upregulation, we used a Ca^2+^ chelator, BAPTA-AM, in our experiments. Our results clearly showed that in the absence of Ca^2+^, the increase in *LMCD1* mRNA levels was abolished in response to AngII treatment (Figure 6A). Additionally, pretreating VSMCs with 50 μM CK59, a calcium-calmodulin-dependent kinase II inhibitor, also resulted in a statistically significant decrease in AngII-mediated *LMCD1* gene expression changes compared to the DMSO-treated control group (Figure 6B). We also assessed the involvement of PKC in the AngII-induced *LMCD1* expression regulation using 1 μM Ro-31-8425, a selective inhibitor of PKC, prior to 100 nM AngII stimulation. As shown in Figure 6C, the PKC inhibitor slightly but not significantly blocked the effect of AngII on *LMCD1* expression. Additionally, pretreatment of the vascular smooth muscle cells with 1 μM PF-562271, an inhibitor of calcium-dependent proline-rich tyrosine kinase 2 (PYK2), did not significantly reduce the AngII-induced upregulation of *LMCD1* (Figure 6D). From these observations, we conclude that AT1-R activation leads to *LMCD1* upregulation mainly through G_q/11_-regulated Ca^2+^ signaling with the involvement of calcium-calmodulin-dependent CaMKII, but the PKC and PYK2 have no key roles in the regulation of AngII-induced *LMCD1* upregulation. 

### 2.6. Evaluation of MAPK Cascade-Dependent Transcriptional Effects in LMCD1 Expression Regulation

**Figure 7:**
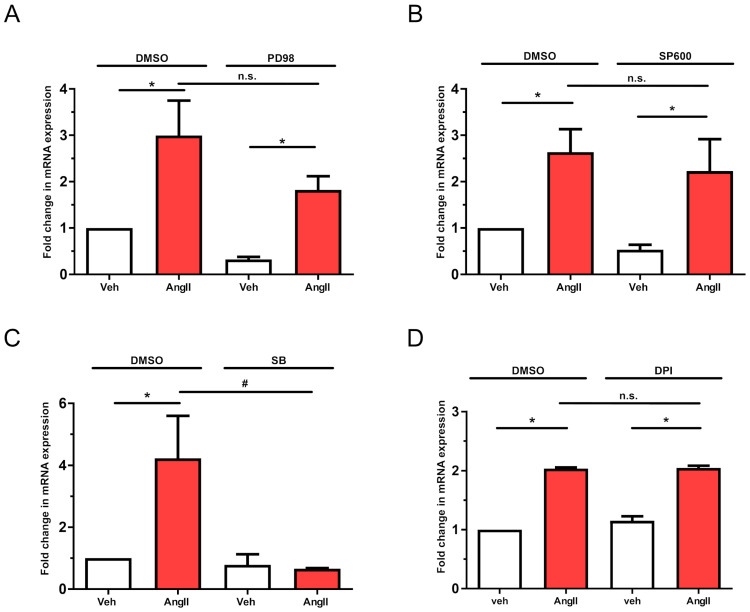
Involvement of MAPK cascade activation in AngII-induced upregulation of *LMCD1*. VSMCs were serum-deprived overnight and then pretreated for 30 min with either DMSO as a control or with one of the following inhibitors: (**A**) 20 µM MEK inhibitor PD98059 (PD98), (**B**) 25 µM JNK inhibitor SP600125 (SP600), (**C**) 50 µM p38 MAPK inhibitor SB202190 (SB), or (**D**) 5 µM diphenyleneiodonium chloride (DPI). Following pretreatment, VSMCs were stimulated with either vehicle (white columns) or 100 nM AngII (red columns) for 2 h. mRNA samples were prepared and transcribed to cDNA, and *LMCD1* expression levels were measured by qRT-PCR and normalized to the *GAPDH* housekeeping gene. Data are presented as values normalized to DMSO vehicle samples (mean ± S.E.). Statistical significance was determined using multiple linear regression, with *p* < 0.05 considered to be statistically significant. Significance compared to vehicle stimulation is indicated by *; significance compared to DMSO-pretreated agonist-induced response is indicated by #; while n.s. indicates not statistically significant (*p* > 0.05). Values represent three to five independent experiments (*n =* 3–5).

We investigated the roles of the ERK1/2, p38, and JNK MAPK pathways in AT1-R-mediated regulation of *LMCD1* gene expression. Pretreatment with the MEK inhibitor PD98059 or the JNK inhibitor SP600125 did not significantly reduce AngII-induced upregulation of *LMCD1* (Figure 7A,B). Previous studies have shown that p38 MAPK activation upregulates *LMCD1* expression in cardiomyocytes [19]. To explore whether this occurs in other cardiovascular cells, we examined vascular smooth muscle cells by inhibiting p38 MAPK with SB203580. As shown in Figure 7C, p38 MAPK inhibition significantly decreased AngII-induced *LMCD1* upregulation.

It is also known that MAPK activation can be driven by reactive oxygen species (ROS) [24]. For ROS production, activation of NADPH and NADH oxidases (NOXs) is required, and it is established that NOX isoforms can be activated by AT1-R signaling [25]. To clarify the role of ROS in AngII-dependent *LMCD1* upregulation, we used the NOX inhibitor diphenyleneiodonium (DPI). Our results indicate that 5 µM DPI pretreatment did not affect AngII-induced *LMCD1* expression changes, suggesting that ROS-mediated MAPK activation does not play a role in this process (Figure 7D).

### 2.7. Effects of LMCD1 Overexpression on Cellular Functions

**Figure 8:**
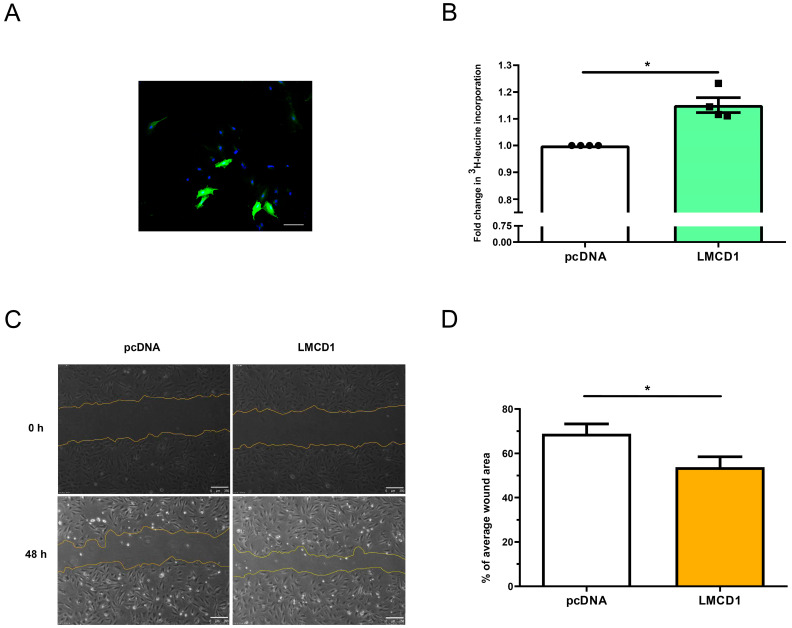
Effects of LMCD1 on VSMC functions. (**A**) A7R5 cells were transfected with LMCD1-overexpressing plasmids, fixed with paraformaldehyde, and stained using an anti-LMCD1 primary antibody followed by an AlexaFluor488-labeled secondary antibody (green). Cell nuclei were counterstained with To-Pro reagent (blue). Immunofluorescent signals were analyzed using a Zeiss LSM710 confocal laser-scanning microscope. Scale bar represents 100 µm (**B**–**D**) A7R5 cells were transfected with either empty pcDNA3.1 plasmids or LMCD1 overexpressing plasmids. (**B**) Cell proliferation was measured using a ^3^H-leucine incorporation assay. Transfected cells were incubated with ^3^H-leucine for 24 h, then samples were collected, and scintillation counts were measured. Values were normalized to the empty vector control values. Data are presented as mean ± S.E. (Empty pcDNA3.1—white column; LMCD1 overexpressing—teal column). Statistical significance was determined with a paired *t*-test (* *p* < 0.05). Data represent four independent experiments *(n =* 4). (**C**,**D**) Cell migration was assessed with a wound-healing assay. (**C**) Wounds were created with a sterile pipette tip in the cell culture wells, and images were captured immediately after the scratch and after 48 h at 5× magnification, following the measurement of the area of the wound. (**C**) Representative images of the wound areas. Scale bars represent 250 µm. (**D**) Quantification of wound areas as a percentage of the average wound area at the 0-h time-point (Empty pcDNA3.1—white column; LMCD1 overexpressing—yellow column). Statistical significance was determined with a paired *t*-test, with *p* < 0.05 considered to be statistically significant (* *p* < 0.05). The values are from three to four independent experiments (*n* = 3–4).

After characterizing the AngII-activated signaling pathways leading to *LMCD1* upregulation, we examined the effects of LMCD1 on the physiological functions of vascular smooth muscle cells. Due to the challenges in transfecting primary rat VSMCs, we used the immortalized rat A7r5 vascular smooth muscle cell line for LMCD1 overexpression using pcDNA3.1 plasmid constructs. Control cell groups were transfected with empty pcDNA plasmids while LMCD1-overexpressing cells were transfected with pcDNA plasmids containing *LMCD1* DNA. Microscopy images revealed that, on average, 18–28% of the cells expressed LMCD1 protein 48 h after transfection (Figure 8A).

To assess the impact of LMCD1 overexpression on cell proliferation, we conducted a ^3^H-leucine incorporation assay. One day after the transfection, cells were incubated in a ^3^H-leucine-labeled medium for 24 h, followed by scintillation counting. As shown in Figure 8B, LMCD1-overexpressing cells exhibited a significant increase in radioactive scintillations compared to control cells transfected with empty pcDNA plasmids. This enhanced incorporation of labeled leucine suggests that LMCD1 promotes increased cell proliferation, supporting previously documented findings on the proliferative effect of LMCD1.

The increase in cellular migration is an important phenomenon in vascular remodeling. To investigate the impact of LMCD1 on cellular migration, we used a wound-healing assay. Twenty-four hours after the transfection with LMCD1-expressing or empty pcDNA3.1 plasmids, a wound was scraped in the cellular layer with a sterile pipette tip. Wounds were photographed at marked positions immediately after scratching and again 48 h later. In Figure 8C, LMCD1-overexpressing cells demonstrate a significantly increased cell migration capacity: after 48 h, the wound area in LMCD1-overexpressing cells decreased to 54% compared to 69% in control cells (Figure 8D).

## 3. Discussion

The long-term effects of excessive activity of the RAAS are well-known contributors to the pathogenesis of various cardiovascular diseases, including hypertension and atherosclerosis. The exaggerated activation and effects of AT1-R in vascular smooth muscle cells play a critical role in the formation of these pathological and often lethal conditions by inducing gene expression changes that lead to vascular remodeling processes such as proliferation, cell migration, and fibrosis [6]. While many AngII-upregulated proteins in VSMCs have been linked to vascular remodeling [26], the full understanding remains incomplete. Here, in this study, we focused on LMCD1 (formerly also known as Dyxin), a protein that has already been described to have proliferative effects in various cell types [19,27,28,29], whose role in the physiology of VSMCs and association with AngII-induced signaling pathways has not been clearly characterized.

We performed an Affymetrix GeneChip Microarray Expression Analysis to explore gene expression changes mediated by AngII stimulation, aiming to identify novel genes that are significantly regulated by this hormone and might play a role in AngII-induced vascular effects. As shown in Figure 1A, the volcano plot from the transcriptome assay indicates that *LMCD1* is one of the genes that are significantly upregulated in response to 100 nM AngII stimulation. Given that the LMCD1 protein has already been described to have proliferative effects in many tissues, including cardiovascular tissues and cells, we sought to investigate its AngII-related upregulation and functional characteristics. 

Next, we aimed to investigate the time course of *LMCD1* gene transcription and LMCD1 protein expression in VSMCs in response to AngII stimulation. As shown in Figure 2A, the increase in *LMCD1* gene expression becomes significant one hour after the AngII stimulation and starts to decrease after five hours. Examination of protein expression revealed that LMCD1 level is highest around 24 h after the AngII stimulation, with levels decreasing after 48 h (Figure 2B,C). Based on these results, we decided to use 24-h AngII stimulation for experiments concerning LMCD1 protein expression, and we used 2-h simulation for experiments investigating *LMCD1* gene expression. 

Immunofluorescence microscopy in Figure 2C suggested a Golgi and nuclear localization for the LMCD1 protein. Labeling different cell compartments with specific markers confirmed our hypothesis: LMCD1 protein showed colocalization with both cis- and trans-Golgi apparatus markers and was also present in the nucleus (Figure 3). Given that LMCD1 primarily acts as a transcriptional co-factor, its nuclear localization aligns with its function. Although LMCD1’s presence in the Golgi apparatus has not been reported, further studies are needed to explore whether the protein is merely stored in the Golgi or serves a specific function there. 

To elucidate the signaling pathways leading to the AngII-induced upregulation of *LMCD1*, we performed experiments using various pharmacological inhibitors and measured gene expression changes with a quantitative real-time PCR method. Given that AngII (or its metabolites) can bind different receptors on VSMCs, we aimed to identify the receptor primarily responsible for *LMCD1* upregulation. Using candesartan, a highly specific AT1-R inhibitor, our results indicated that AT1-R plays a dominant role in AngII-mediated *LMCD1* expression increase (Figure 4A). While AT1-R is mainly known for its G_q/11_-coupled signal transduction, it also engages other G proteins, such as G_12/13_, and small G proteins, as well as G-protein-independent signaling pathways involving β-arrestin recruitment are also important [2]. We examined which of these pathways might be involved in LMCD1 regulation. In our approach, we first pretreated VSMCs with YM-254890, a G_q/11_ protein inhibitor, prior to AngII stimulation (Figure 4B). In separate experiments, we stimulated the cells with TRV120023, a β-arrestin-biased agonist of AT1-R (Figure 4C). Finally, we examined the effect of AVP, as its V1 vasopressin receptor on VSMCs is also primarily linked to G_q/11_ (Figure 4D). As shown in Figure 4B, blocking G_q/11_ completely abolished AngII-mediated *LMCD1* gene expression changes. Additionally, AVP stimulation also increased *LMCD1* expression, though to a lesser extent than AngII (Figure 4D). By contrast, stimulation of VSMCs with the biased agonist TRV120023 did not significantly alter *LMCD1* expression (Figure 4C). These observations exclude the importance of β-arrestin-mediated signaling in gene expression regulation of *LMCD1* and confirm that LMCD1 upregulation occurs via a G_q/11_-initiated mechanism in response to AngII stimulation in VSMCs.

In contrast to our previous studies investigating other gene expressions [30], AngII-induced regulation of *LMCD1* gene expression was not dependent on EGFR transactivation. As shown in Figure 5A, neither 50 ng/mL EGF stimulation (alone or combined with AngII) nor pretreatment of cells with growth factor receptor inhibitors significantly affected the AngII-induced *LMCD1* upregulation (Figure 5B–E).

The alpha subunit of the activated G_q/11_ protein activates phospholipase Cβ, which hydrolyzes PtdIns(4,5)*P*_2_ to produce DAG, an activator of PKC, and Ins(1,4,5)*P*_3_, an agonist of ER Ca^2+^ channels. An increase in cytosolic calcium concentration in VSMCs leads to the activation of various Ca^2+^-dependent kinases [1,31]. Pretreatment of VSMCs with Ca^2+^ chelator BAPTA-AM, as well as pharmacological inhibitors of CaMKII, PKC, and Pyk2, revealed that calcium signal plays a crucial role in the AngII-induced LMCD1 level increase (Figure 6A), whereas PKC and Pyk2 activities do not significantly affect this process (Figure 6C,D). In contrast, the inhibition of CaMKII significantly reduced the AngII-induced upregulation of LMCD1 (Figure 6B), though this effect was not as pronounced as that observed with BAPTA-AM. These findings suggest that parallel pathways may also be involved in the signaling leading to *LMCD1* upregulation. 

Additionally, we investigated the roles of different MAPK isoforms known to participate in AngII-mediated gene expression changes. Our results clearly demonstrated that p38 MAPK inhibition significantly reduces the effect of AngII on *LMCD1* expression (Figure 7C). Inhibiting the MEK-ERK1/2 and JNK pathways revealed that they do not have significant relevance in this process (Figure 7A,B). AT1-R-mediated p38 MAPK activation can occur through various mechanisms. For instance, AngII-related reactive oxygen species (ROS) production, which is linked to NADPH oxidase (NOX) activation, plays an important crucial role in vascular functions, including remodeling and other pathological consequences [30,31]. However, our experiments using diphenyleneiodonium chloride (DPI), a commonly used pan-NOX-inhibitor, indicated that ROS production is not significantly involved in *LMCD1* upregulation in VSMCs (Figure 7D). p38 MAPK activation may also occur via non-receptor tyrosine kinases, such as Src family member kinases, focal adhesion kinase (FAK), and Pyk2 [32,33]. However, pretreatment with Src inhibitor SKI-1 (Figure 7D), as well as inhibition of FAK and Pyk2 (Figure 6D), did not significantly affect AngII-induced *LMCD1* expression. Ca^2+^-dependent CaMKII activation can serve as an upstream regulator of p38 MAPK [34,35,36] since CaMKII inhibition effectively reduces the AngII-mediated *LMCD1* upregulation (Figure 6B). Taken together, it appears that p38 MAPK activation involves the CaMKII pathway, but other mechanisms are possible due to the incomplete effect of CaMKII inhibition. 

After investigating the AngII-mediated signaling pathways that lead to increased *LMCD1* expression, we examined potential changes in vascular functions associated with increased LMCD1 levels. To evaluate the functional attributes of LMCD1 in vascular smooth muscle, we overexpressed the LMCD1 protein in an immortalized vascular smooth muscle cell line, A7r5, as the rat primary VMCSs proved challenging to transfect. Despite the fact that the transfection efficiency of the cells was only 18–28%, the results of our functional assays showed notable effects. Cell proliferation was assessed using a ^3^H-leucine incorporation assay (Figure 8B), and it was demonstrated that even partial LMCD1 overexpression led to increased cellular protein synthesis, a marker strongly associated with cell proliferation. Enhanced cell migration is another important aspect of vascular remodeling. This was assessed with an in vitro wound-healing scratch assay, and we examined the migration intensity of LMCD1 overexpressing A7r5 cells (Figure 8C). Forty-eight hours after scratching, LMCD1-overexpressing cells showed significantly higher cell migration rates compared to controls, reinforcing the role of LMCD1 in VSMC proliferation and cell migration, supporting the data that LMCD1 is an important factor in the regulation of VSMC proliferation and migration [37]. This study did not investigate whether AngII-regulated LMCD1 functions as a transcription factor. Our in vitro observations in VSMCs revealed that LMCD1 is localized to the Golgi apparatus, with slightly stronger signal intensity compared to the nucleus. This finding suggests that LMCD1 may have regulatory functions beyond transcriptional control. Further studies are required to explore this possibility and to delineate both the transcriptional and non-transcriptional roles of LMCD1 in vascular cells. Additionally, the precise involvement of LMCD1 in AngII-induced vascular changes needs to be validated through in vivo studies.

## 4. Materials and Methods

### 4.1. Materials

Plates and dishes for cell cultures were obtained from Greiner (Kremsmunster, Austria). Unless otherwise mentioned, all cell culture and molecular biology reagents were purchased from Thermo Fischer Scientific (Waltham, MA, USA). AngII, EGF, AVP, AG1478, AG1024, AG538, BAPTA-AM, CK59, PD98058, PF-562270, and SP600125 were ordered from Sigma-Aldrich (St. Louis, MO, USA). YM-25489 was purchased from Wako-Chemicals (Neuss, Germany). TRV120023 peptide (Sar-Arg-Val-TYR-Lys-His-Pro-Ala-OH) was synthesized by Proteogenix (Schiltigheim, France) to more than 98% purity. The anti-LMCD1 antibody was obtained from Abcam (Cambridge, UK), anti-β-actin antibody was purchased from Sigma-Aldrich (St. Louis, MO, USA), HRP-linked secondary antibodies were purchased from Cell Signaling Technology (Danvers, MA, USA), fluorescent antibodies for Western blot assay were purchased from Azure Biosystems (Dublin, CA, USA) and fluorescent secondary antibodies for immunocytochemistry were obtained from Thermo Fisher Scientific (Waltham, MA, USA). FastStart Essential DNA Green Master Mix was purchased from Roche (Basel, Switzerland). Unless otherwise mentioned, other chemical reagents were obtained from Sigma-Aldrich (St. Louis, MO, USA).

### 4.2. Isolation of Primary VSMCs

The thoracic aorta of 40–60-day-old male Wistar rats (170–250 g) were used for cell culture preparation (Charles River Laboratories-Semmelweis University, Budapest). All animal procedures were approved by the Animal Care Committee of the Semmelweis University, Budapest, and by Hungarian authorities (No. 001/2139-4/2012), following legal and institutional guidelines for animal care. The investigation complies with the Guide for the Care and Use of Laboratory Animals (NIH, 8th edition, 2011).

The rat VSMCs were isolated and cultured according to the standard explant method [38]. Briefly, the animals were sacrificed by decapitation and fast bleeding. The thoracic aorta was removed and carefully cleaned from adherent fat, connective tissues, and blood. The prepared aorta was cut into small pieces and treated with collagenase. After digestion, the small pieces were placed on a sterile plate in DMEM medium (Biosera, Nuaille, France) supplemented with 10% FBS (Biosera, Nuaille, France), 1% Glutamax (Gibco, Dublin, Ireland) and 1% penicillin-streptomycin (Lonza, Gampel, Switzerland). VSMCs were allowed to grow at 37 °C for 7–14 days, then passaged 2–3 times. Experiments were typically carried out after the third passage. The isolated VSMCs exhibited normal responses to Ang II stimulation, such as calcium signaling and ERK activation, and the homogeneity of primary VSMC cultures was checked by smooth muscle alpha-actin immunostaining [28].

### 4.3. Cell Cultures

Most experiments were conducted on primary VSMCs isolated from young male Wistar rats. Additional experiments were performed on A7r5 cells, an immortalized rat vascular smooth muscle cell line obtained from the American Type Culture Collection (ATCC, Manassas, VA, USA). The A7r5 cells were subcultured in DMEM medium (Biosera, Nuaille, France) supplemented with 10% FBS (Biosera), 1% penicillin-streptomycin (Lonza, Gampel, Switzerland), and 1% Glutamax (Gibco, Dublin, Ireland). Cells were seeded onto 6-well plates and cultured to approximately 90% confluency at the final passage. A total of 16–24 h before the experiment, the medium was changed to serum-free DMEM. All cell cultures were stored at 37 °C in a 5% CO_2_ atmosphere.

### 4.4. Treatment Protocols

Before experiments, VSMCs were serum-deprived for 16–24 h, followed by treatment with agonists (AngII, AVP, EGF, or TRV120023), typically for 2 h. Control cell groups were treated with serum-free DMEM solution (vehicle). For time-dependence measurements, AngII stimulation was applied for 1 h to 6 h. In experiments involving inhibitors, VSMCs were pretreated with specific compounds or dimethyl sulfoxide (DMSO) for 10 or 30 min before stimulation with AngII, AVP, EGF, or TRV120023 for 2 h, alongside a control group stimulated with vehicle (serum-free DMEM). 

### 4.5. DNA Construct

A DNA plasmid construct was designed to express HA-tagged LMCD1 protein using the pcDNA3.1 backbone. To amplify the full ORF of LMCD1, cDNA from VSMCs stimulated with AngII for 2 h served as the template. The initial PCR product was separated by electrophoresis, purified using the GeneJet Gel Extraction Kit (Thermo Fisher Scientific), and then subjected to a second round of PCR with primers containing restriction enzyme sites and the HA-tag sequence.

### 4.6. Plasmid Transfection

A7r5 cells were used for LMCD1 overexpression using pcDNA3.1 plasmid constructs. For the wound-healing assay, a total of 200,000–250,000 A7r5 cells were seeded into 6-well plates. In the case of the 3H-leucine incorporation assay, 20,000–30,000 A7r5 cells were passaged on 24-well plates one day before transfection. The cells were transfected using Lipofectamine 2000 Transfection Reagent (Thermo Fischer Scientific, Waltham, MA, USA), following the manufacturer’s instructions. Cells were used for experiments at least 24 h after the transfection. 

### 4.7. Affymetrix GeneChip Analysis

Details of the analysis of the Affymetrix GeneChip raw data analysis are described in a previous study [30]. Briefly, serum-starved VSMCs were stimulated with either vehicle or 100 nM AngII for 2 h at 37 °C, after which cells were lysed in Trizol reagent to prepare RNA. The analysis was conducted on the Affymetrix Rat Gene 1.0 ST GeneChip Array (Affymetrix, Santa Clara, CA, USA) by UD-GenoMed Medical Genomic Technologies Ltd., University of Debrecen, Debrecen, Hungary. Microarray experiments were performed in triplicate. Raw CEL files were background-corrected and normalized using the *oligo* R package (1.58.0), and differential expression (AngII vs. vehicle) was analyzed with the *limma* R package (3.50.3). Pathway activities were inferred from log2 fold-changes in the differential gene expression profile using the *decoupleR* Python package (1.2.0). Weighted interactions between pathways and genes were obtained from PROGENy. A multivariate linear model was fitted that predicts the observed gene expression based on the PROGENy pathway-gene interactions. The t-values of the slopes reflect the activity score. A positive score indicates an active pathway, while a negative indicates inactivity.

### 4.8. RNA Isolation and cDNA Preparation

To isolate total RNA from VSMCs, the treatment of the cells was stopped by removing the medium and by washing the cells twice with cold, sterile PBS (137 mM NaCl; 2.7 mM KCl 2.7; 10.1 mM Na_2_HPO_4_; 1.8 mM KH_2_PO_4_, pH 7.4). Total RNA was extracted using the RNeasy Plus Mini kit (Qiagen, Hilden, Germany) following the manufacturer’s instructions. RNA concentrations were measured spectrophotometrically by absorbance at 260 nm, and purity was assessed by the 260/280 and 230/260 nm ratios using a NanoDrop OneC spectrophotometer (Thermo Fischer Scientific, Waltham, MA, USA). Prior to the quantitative real-time PCR measurement, total RNA was reverse-transcribed to cDNA using the RevertAid Reverse Transcription Kit (ThermoFisher Scientific), according to the manufacturer’s instructions.

### 4.9. Quantitative Real-Time PCR

Gene expression changes were measured using a quantitative real-time PCR method. LightCycler 480 SYBR Green I Master kit (Roche, Basel, Switzerland) was used for the PCR reaction, following the manufacturer’s instructions, and a LightCycler 480 system (Roche, Basel, Switzerland) was used for the measurements. To determine mRNA levels of LMCD1, relative quantification mode was used with glyceraldehyde-3-phosphate dehydrogenase (*GAPDH*) as the reference housekeeping gene. The following primers were used for qRT-PCR determinations (5′-3′): GAPDH: Forward CCT GCA CCA CCA ACT GCT TAG, Reverse CAG TCT TCT GAG TGG CAG TGA TG; LMCD1 Forward CCT CGA GTG CAA AAG ATG TCC, Reverse AAT TTT CCG ATC ATC CTC CA. The thermal cycling program started with a 5-min pre-incubation at 95 °C, followed by 45 cycles of amplification. Each cycle consisted of 10 s at 95 °C, 5 s at 62 °C, and 15 s at 72 °C. Amplification was followed by a melting curve starting at 95 °C for 5 s, then 1 min at 65 °C and 97 °C, and cooling for 30 s at 40 °C. Fluorescence data, including melting curves, were obtained. Cycle threshold (Ct) values were calculated by the second derivative method using the advanced relative quantification analysis mode of LightCycler 480 Software 1.5.1. *GAPDH* was used for normalization, and gene expression levels were plotted against *GAPDH* expression. To calculate fold-changes in gene expression, the following formula was used: Ratio = E^ΔCt target gene^/E^ΔCt GAPDH^.

### 4.10. Western Blot Analysis

Samples for Western blot analysis were scraped into SDS sample buffer, supplemented with phosphatase and protease inhibitors, and then sonicated. Protein samples were separated on SDS-polyacrylamide gels and then transferred to PVDF membranes. After transfer and blocking, membranes were incubated with specific primary and secondary antibodies, some of which were HRP-labeled and visualized with chemiluminescent substrate reagents (Immobilion Western HRP substrate reagent, Millipore, Billerica, MA, USA), while other antibodies were labeled with fluorescent molecules. Both chemiluminescent and fluorescent signals were detected with the Azure c600 system (Azure Biosystems, Dublin, CA, USA). Results were quantified by densitometry with the help of ImageJ software 1.53e. 

### 4.11. Immunocytochemistry

VSMCs were plated onto 8-well ibidi-plates (10,000 cells/well) and then stimulated with 100 nM AngII after overnight serum deprivation. Cells were fixated with 4% paraformaldehyde diluted in PBS and blocked with 5% BSA. Thereafter, cells were stained with different primary antibodies followed by fluorescent secondary antibodies (Alexa Fluor 488 or Alexa Fluor 568; Thermo Fischer Scientific). Nuclei were labeled with To-Pro reagent (Invitrogen, Waltham, MA, USA). Images were captured using a Zeiss LSM 710 confocal microscope (Zeiss AG, Jena, Germany).

### 4.12. ^3^H-Leucine Incorporation Assay

A7r5 cells were seeded on a 24-well plate (20,000–30,000 cells/well) and then transfected with LMCD1 overexpressing pcDNA3.1 plasmid or empty pcDNA3.1 plasmid. The next day, transfected cells were labeled with tritium-labeled leucine in serum-free DMEM for 24 h. Samples were collected by the following protocol: Cells were washed twice with ice-cold PBS and treated with 5% trichloroacetic acid (TCA) on ice. After 30 min, TCA was removed, and wells were washed twice with room-temperature PBS. Next, 0.5 mL 0.5 M NaOH was pipetted to the wells for 30 min at room temperature, and samples were collected into 10 mL of OptiPhase HiSafe3 scintillation cocktail (PerkinElmer, Waltham, MA, USA) containing vials. Wells were additionally washed with 100 µL of distilled water, which was also added to the vials. The radioactivity values were determined in a liquid scintillation counter, with a control sample containing only distilled water in the scintillation cocktail. 

### 4.13. Wound-Healing Assay

To assess the migration capacity of cells, a wound-healing assay was performed. A sterile P200 pipette tip was used to scrape the cell monolayer, creating a wound. The medium was then replaced with DMEM containing 2% FBS to minimize proliferation-related effects. The wounded areas were photographed immediately after scratching using a Leica DMI6000 B (Leica, Wetzlar, Germany) microscope at 5× magnification. After 48 h, the same areas were re-photographed under identical conditions. Cell migration was quantified by analyzing the images with ImageJ software 1.53e.

### 4.14. Statistical Analysis

Gene expression data from qRT-PCR measurements were analyzed using multiple linear regression with a 95% confidence interval in order to determine the significance of inhibitor treatments, stimuli, and their interactions on the fold-change value of a given gene of interest. In the case of Figure 2, Figure 4B,C and Figure 7, one-way ANOVA analyses were performed to compare stimulated and control groups. For the evaluation of ^3^H-leucine incorporation assay and wound-healing assay (Figure 8), paired *t*-tests were used. Statistical analyses and graph plotting were carried out with GraphPad Prism 9.1.2 software. Sample size (*n*) in the figure legends refers to the number of independent experiments (biological replicates). Unless otherwise stated, data are presented as mean ± SE.

## 5. Conclusions

**Figure 9:**
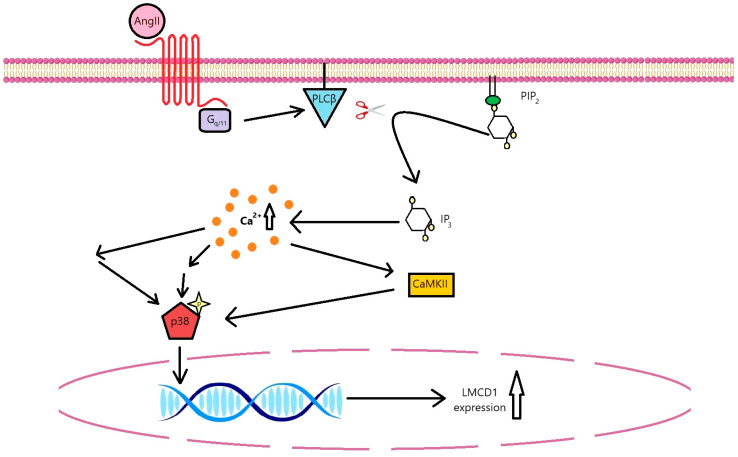
Proposed signaling pathway in VSMCs in response to AngII stimulation leading to *LMCD1* upregulation. AngII induces *LMCD1* upregulation via AT1-R activation, mediated through G_q/11_ protein coupling. The α subunit of G_q/11_ activates phospholipase-C β (PLCβ), which cleaves phosphatidylinositol 4,5-bisphosphate (PIP_2_) to diacylglycerol and inositol 1,4,5-trisphosphate (IP_3_). IP_3_ triggers Ca^2+^ release from the endoplasmic reticulum, leading to activation of the p38 MAPK pathway probably via multiple mechanisms, including calcium/calmodulin-dependent protein kinase II (CaMKII) activation. The p38 MAPK pathway ultimately drives the upregulation of *LMCD1* in the nucleus.

Taken together, these results reveal that LMCD1 contributes to the vascular response to AngII. LMCD1 upregulation occurs via AT1-R activation and G_q/11_ protein coupling. Calcium signal-induced p38 MAPK activation is the key regulatory mechanism in the upregulation of LMCD1 (Figure 9). Other mechanisms may also act through increased LMCD1 expression, as supported by the literature. Additionally, the elevated expression of LMCD1 enhanced the cell proliferation and migration in VSMCs in in vitro experiments. Our in vitro data suggest that LMCD1 could be a promising pharmacological target in cardiovascular dysfunctions associated with AT1-R overactivation; however, the in vivo relevance of our findings warrants further investigation.

## Data Availability

The datasets generated during the current study are available from the corresponding authors upon reasonable request. Microarray data have been deposited in the ArrayExpress database at EMBL-EBI www.ebi.ac.uk/arrayexpress under accession number E-MTAB-14626 (www.ebi.ac.uk/biostudies/arrayexpress/studies/E-MTAB-14626) (accessed on 15 November 2024).
